# Surgical Diseases of Women—Uterine Polypi

**Published:** 1864-08

**Authors:** J. F. Miner


BUFFALO

### Medical and Surgical Journal.

VOL. IV. AUGUST, 1864. No. 1.

### ORIGINAL COMMUNICATIONS.

ART. I.—*Surgical Diseases of Women—Uterine Polypi.* By J. F. Miner, M. D.

Uterine Polypi are of various forms, and differ greatly in location and structure, and are attended by a great variety of general and local symptoms; their physical and pathological characters are very well understood, while the causes of their growth are obscure and undetermined. A brief description will be premised, the chief object being to indicate the surgical treatment required for removal of these morbid growths.

It is common to arrange these growths under one of three or four different heads, and authors generally give [descriptions of glandular, cellular. fibrous, mucous, and some other varieties of polypi. It is, however, probable that nearly all growing from mucous membranes consist of fibrocellular tissue, infiltrated by serous or synovia-like fluid, the abundance or scarcity of the fluid, contained in the meshes, making the principal difference between the tougher, fibrous or fassia-like, and the softer, mucous or gelatinous varieties. Some of these growths are composed almost wholly of serous or synovia-like fluid, while others contain little, if any. Neither the very soft or very dense contain blood-vessels in abundance, while those not so gelationous or fibrous often contain blood-vessels in some degree, and from this circumstance have been called *vascular*. This form of out-growth is rare in the uterus, and is usually of small growth. Most uterine polypi are feebly supplied with blood-vessels, and many times not any can be positively traced. In size they vary from a line in diameter to that of several inches, and the shape is also varied, though the smooth rounded variety attached by a pedicle of greater or less proportional diameter, is the more common form of the larger growths.

Polypi may be attached to the fundus, walls, inner surface of cervix, or lips of the os-uteri, and upon this will depend the ease and safety with which they may be removed. The point of attachment is sometimes uncertain, a long pedicle may reach much higher than it can be traced. Polypi which grow from the lips or cervical canal do not neccessarily have any pedicle at all, but rest upon the surface by a broad base. Small polypoid growths from the size of a millet seed to that of a pea, often rest upon the lips of the os-uteri, or in the cervical canal, and give rise to troublesome and dangerous hemorrhage, and other distressing symptoms. These growths, if soft and gelatinous are easily removed, while if dense and fibrous, greater, difficulties will be presented.

*General Symptoms.*—The symptoms which attend this disease are such as to rarely mislead, though it must be confessed that sometimes the diagnosis cannot be positive. At first there is often a profuse secretion of mucous, which becomes gradually more abundant, and soon is observed to be more or less mixed with blood; sometimes large quantities of blood will be discharged without any mucous; hæmorrhage is an important symptom, and rarely absent. The smaller growths which sometimes appear in great numbers upon the uterine neck and extend within the cervical canal, often give rise to profuse hemorrhage. There are almost always present constitutional smyptoms of uterine disturbance; the nervous system suffering most severly. When the tumor has become large, or the uterus has enlarged from the disturbance produced by the presence of an intra uterine growth, there is always bearing down pains; these pains sometimes appear almost like labor, coming on in paroxysms, and having the same expulsive effort. When the tumor has attained considerable size the uterus does often take on the same effort as in labor, and the tumor is thus often expelled or pushed as low as possible from the length of the pedicle and place of attachment; it is thus made apparent at the os-uteri when previously its presence could not have been demonstrated. The tumor and uterus, from consequent enlargement, may fill the pelvis and interfere with the functions of the bladder and rectum; this is rare, and the smaller varieties are much more often met in practice. These growths sometimes attain considerable size before their presence is mistrusted; this is seen in the fibrous or dense varieties, when there is neither hæmorrhagic or mucous discharge, and before the uterus has commenced any contraction for expulsion of its contents.

When the above mentioned symptoms are present, it is indispensably proper to institute a careful examination with the finger, speculum, uterine sound, &c., to discover, if possible, their causes. Hæmorrhage should always attract immediate attention, though it will often be neglected, the patient looking upon it as some derangement of menstruation and not as a symptom of disease.

*Diagnosis* cannot always be made positive; polypus may exist within the cavity of the uterus and still we are unable to detect it. Sometimes the os will admit the finger for a distance sufficient to distinguish some foreign growth; or possibly, by the aid of the sound, we may be able to discover that a polypus grows from the interior of the uterus. The os uteri may sometimes be dilated so as to admit of more perfect examination; patient, repeated examinations will in most cases lead to quite satisfactory conclusions.

Polypus has been mistaken for, and is to be distinguished from, pregnancy, inversion of uterus, prolapsus uteri, cauliflower excressence, scirrhus, vaginal cystocele and vaginal hernia. It is believed that careful examination will distinguish it from these conditions, though when it is situated high up within the uterus and cannot be reached it may be impossible in its early stages to determine its presence, or the exact nature of the disease. The more common form of uterine out-growth—the mucous polypi of most authors upon the diseases of women—is some times overlooked by the inexperienced, and to this variety of the disease is directed especial attention. It has so frequently been brought to our notice after having existed for weeks or months and escaped detection, that a simple description and the means of treatment hardly appear sufficient to enlist the attention of many who attempt to find the cause of hæmorrhage which has even become alarming, or profuse leucorrhœa, which is continual and often largely mixed with blood. Examination is made by poor light or in a faulty manner, so that the small vesicles often escape notice and the hæmorrhage is ascribed to other and erroneous causes.

Small, pearly elevations, of various sizes, from that of a pin-head to a pea, with very slender pedicle, or without any, and resting upon the surface of the uterine neck or within the cervical canal, is a quite frequent condition. These growths may be few or many in number, may be hard and resisting, or soft and gelatinous. They are covered by mucous membrane, and supplied with only very small blood vessels, though hæmorrhage is an almost constant symptom of their presence. This arises probably from the injection of the uterine neck consequent upon the disease, and springs more from around the tumor, or from the uterus itself, than from the surface of these morbid growths. The object of this article will have been gained if attention is directed by it, to this form of disease of the uterine neck, even though nothing new be advanced and nothing valuable suggested.

What is necessary for removal of these growths and arrest of hæmorrhagic and leucorrhœal discharge? If they are few in number, they are easily grasped by forceps and removed, or excised with scissors. They are often present in great numbers and in such case are exceedingly small; if dense and firm they may be scraped off, or if soft, they easily rupture and the fluid portion escapes, while penciling the surface with nitrate of silver will insure relief; indeed, nitrate of silver ("the universal remedy,") will often be found sufficient to destroy these growths and prevent recurrence; the stronger caustics will effect the same, though it is well to use the milder remedies first.

These growths vary greatly in form, size and density of structure; they are described as gelatinous, cystic, glandular, etc., but it is probable that there are not great differences in primary composition, but that the proportions of the gelatinous and fibro cellular material are largely varied. This may give rise also to some differences in the application and efficiency of remedies or in operative proceedure. This form of disease of the uterine neck gives rise to all the various general symptoms of uterine disorder; if to the symptoms of inflammation of the neck of the uterus we add hæmorrhage we then have the constitutional indications which will be generally present in this form of uterine outgrowth.

The larger growths which are possessed of a higher organization and having a longer or shorter pedicle, are also of frequent occurrence; they give rise to similar general symptoms, and hæmorrhage, which is so constantly present in the former, is often produced also by the latter. These often commence within the uterine cavity and after attaining a certain size are expelled by contractions of the uterus or are protruded as far as the pedicle will allow, in which situation they will often be found, the fundus of the polypus in the vagaina, the pedicle occupying the cervical canal, and having attachments at its upper portion or within the uterine cavity. These larger polypi may be generally removed with safety; grasping them with forceps and twisting the neck with slight tension until the attachments are broken is a favorite mode of removal. Ligature, and gradual strangulation by means of a wire and double cannula are also measures highly recommended, and in proper cases should be adopted. When the pedicle is large or strong, and indeed in all cases, *inflammation* and *hæmorrhage* are to be avoided; shock and absorption of pus are also sources of danger. Removal by ligature and excission, where it can be practiced, is perhaps preferable, since in this we may avoid in great degree these risks and at the same time the delay, and dangers which attend the partial decomposition, where more gradual strangulation is practiced.—The pedical may sometimes be divided with the ecraseur, and in such case hæmorrhage is rarely dangerous, or difficult of control.

One other form of disease, sometimes called polypus, may be mentioned in connection with this brief notice of uterine outgrowth. Gradual accumulation of blood within the uterine cavity, which in course of time passes through changes by which the coloring matter is partially removed from its exterior, while the inner portions are of a dark granular character and having a feeble attachment with very imperfect organization, are occasionally observed. We have met with a few of these bodies, and could at first hardly accept this explanation of their growth. They are separated easily from their attachments and do not give rise to much hæmorrhage; the system however is greatly disturbed by their presence in the uterus. One of these bodies recently removed had given rise to so many of the symptoms of pregnancy that it was regarded as such, and the family physician had watched the case, expecting daily to be called to attend in labor, until the period of gestation had been so greatly extended as to excite some suspicion. "Labor pains" had been present at times for several weeks; "motions of the child distinctly felt;" breasts enlarged and secreting a white fluid; hæmorrhage constant and so profuse as to have greatly reduced the strength. The substance was oval, an inch and a half in diameter, with slender, frail pedicle, attached high up in cervical canal, or within the uterus.

Upon section after removal the growth appeared made up of layers, the sixteenth of an inch in thickness, externally, while the inner portion was softer, red and granular. All symptoms of disease disappeared after removal.

Authors give the causes of these fibrinous accumulations and usually attribute them to conception and attempt at abortion, or to effusions of blood within the uterine cavity in connection with pregnancy or abortion Other explanations are attempted, but it appears sufficiently conclusive that they are produced from effusions of blood into the uterine cavity and subsequent partial organization. They are shaped by the cavity in which they are formed and after partial expulsion may be easily grasped and removed. While retained in the uterus, the exact character could scarcely be determined.

